# Novel Endovascular Technique for Thrombus Removal: The “Super Grab a Clot and Hold ON (Super GACHON)” Technique

**DOI:** 10.1155/cric/5525113

**Published:** 2025-01-09

**Authors:** Takahiro Tokuda, Hideyuki Takimura, Yasuhiro Oba, Keisuke Hirano

**Affiliations:** ^1^Department of Cardiology, Nagoya Heart Center, Nagoya, Aichi, Japan; ^2^Department of Cardiology, Sogo Tokyo Hospital, Tokyo, Japan; ^3^Department of Cardiology, Toyohashi Heart Center, Toyohashi, Aichi, Japan

**Keywords:** case report, GACHON, novel technique, Super GACHON, thrombus

## Abstract

We present a case of a 60-year-old man with claudication in his right foot; the patient had received stent-graft implantation for the right superficial femoral artery (SFA) 1 year ago. Computed tomography angiography suggested stent occlusion of the right SFA, and a thrombus was considered to cause occlusion. To avoid distal embolization, we performed lesion recanalization via a trans-ankle intervention. First, we performed aspiration for the lesion; however, the thrombus persisted. Second, we performed the “Super Grab a Clot and Hold ON” technique and removed several thrombi. Finally, we used drug-coated balloons as a final device for the lesion. Two years later, the right SFA was found open upon an ultrasonography.

## 1. Introduction

Technical and device advancements have improved the clinical outcomes of endovascular treatment (EVT); nonetheless, recanalizing calcified and thrombus lesions is challenging because of the increased risk of vessel perforation, insufficient expansion, distal embolization, and reocclusion [1, 2]. In Japan, an atherectomy device is available only for calcified lesions. By contrast, an aspiration thrombectomy device has not been approved yet. Conventionally, clinicians perform EVT with manual aspiration, ballooning, or stenting and hybrid treatment using a Fogarty catheter to manage thrombi lesions. Shirai, Hirano, and Kobayashi [3] reported on the “Grab a Clot and Hold ON (GACHON)” technique as an EVT method for a thrombus lesion. The GACHON technique is a method in which a thrombus located in the nonstenting zone is moved to a location where stent placement is possible using a S.M.A.R.T. stent (Cordis Endovascular, Warren, NJ, United States), followed by the placement of the stent along with the thrombus. However, its disadvantages are that a stent must be placed finally, and only one opportunity exists to grab a clot. In this case, we utilized a “Super GACHON” technique to overcome the disadvantages of the “GACHON” technique.

## 2. Case Presentation

**Figure 1:**
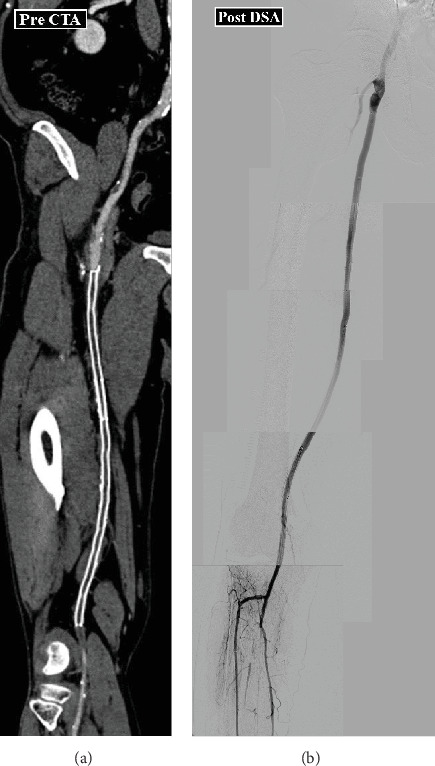
Pre- and postprocedural results. (a) Preprocedural computed tomography angiography. (b) Postprocedural distal subtraction angiography.

A 60-year-old man with severe claudication in his right foot was referred to our hospital for evaluation and treatment. He had a history of hypertension and dyslipidemia and had received intervention for the right superficial femoral artery (SFA) 1 year ago. In the intervention, 6 × 250 and 6 × 150 mm stent grafts (Viabahn; WL Gore, Flagstaff, AZ, United States) were implanted for his right SFA. The ankle-brachial index (ABI) of his right foot was 0.45. Computed tomography (CT) angiography suggested stent occlusion of the right SFA, and all below-the-knee arteries were patent; a procedure for lesion recanalization was scheduled (Figure 1(a)). A thrombotic lesion was considered with the CT finding; therefore, we planned EVT for the lesion via a trans-tibial intervention to avoid distal embolization after ballooning. A 6 Fr sheath (Parent select; Medikit Corp., Bunkyo, Tokyo, Japan) was inserted from the dorsal artery into the popliteal artery. Heparin 5000 IU was administered intra-arterially and activated; the clotting time was maintained at > 250 s. First, we used a guiding catheter (Mach one; Boston, Marlboro, CM, United States) to aspirate the thrombotic lesion; however, it did not work. Therefore, we decided to perform a “Super GACHON” technique.

**Figure 2:**
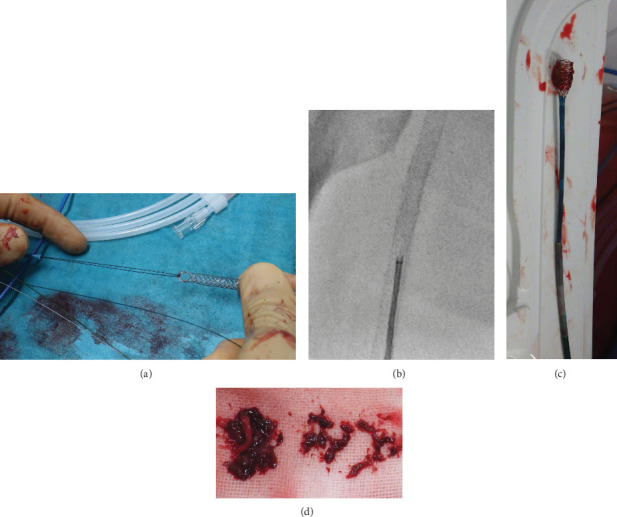
Steps to use the “Super GACHON” technique. (a) Constructing a handmade manual thrombus removal device. (b) Pulling out one-half of the Supera stent and the entire guiding catheter back from the proximal superficial femoral artery. (c, d) A large amount of thrombus is attached to the Supera stent; it has been removed from the body. GACHON: Grab a Clot and Hold ON.

First, the interwoven nitinol stent (Supera; Abbott, Chicago, IL, United States) alone is prepared outside the body without placing it inside the vessel. In this case, we used a 6 × 40 mm Supera stent. Second, the 300 mm guidewire (Jupiter FC; Boston, Marlboro, CM, United States) was folded in half and hooked to the edge of the stent. The guidewire was placed inside the Mach1 guiding catheter (Figure 2(a)). Next, the guiding catheter with the Supera stent was advanced to the proximal site of occlusion without a guidewire (Figure 2(b)). With one-half of the Supera stent exposed, the guiding catheter was pulled back, and a clot was grabbed (Figure 2(c)). With this technique, clinicians do not need to deploy the Supera stent and can use the guiding catheter with the Supera stent several times. In this case, we used the handmade manual thrombus removal device five times and removed numerous thrombi (Figure 2(d)). Based on the angiography and intravascular ultrasound findings, we confirmed that the thrombi had disappeared and could proceed to the next step.

After this maneuver, we inserted a guidewire easily and dilated the lesion with a 6.0 mm balloon. We used 6 mm drug-coated balloons for the lesion as a final device without stent deployment and procedural complications (Figure 1(b)). After the intervention, his symptoms improved, ABI increased to 1.03, and primary patency was achieved for up to 2 years.

Informed consent has been obtained from the patient for publication of the case report and accompanying images.

## 3. Discussion

Here, we present a case of in-stent occlusion treated with a novel technique, namely, the “Super GACHON” technique. In this case, we developed two important treatment strategy points.

First, a thrombotic lesion was considered as the cause of occlusion and the occlusion length was > 30 cm. Therefore, the risk of distal embolization may have increased. A report of a manual distal protection device suggested a 93% rate of preventing distal embolization, thereby indicating that a distal protection device did not necessarily prevent distal embolization [4]. This phenomenon may be attributed to the movement of the protection device or patient during catheter manipulation.

By contrast, retrograde tibial access has an increased possibility of preventing distal embolization [5]. We selected a dorsal artery as the access route to prevent distal embolization.

Second, we used a handmade manual thrombus removal device, termed the “Super GACHON” technique. Compared with the “GACHON” technique, the advantages of this technique are that it does not require final stent deployment and could be used numerous times to remove thrombus. Additionally, we used a Supera stent because the finer the stent mesh, the better the ability to remove blood thrombus.

Therefore, we removed numerous thrombi with this handmade device. The catheter intervention for thrombotic lesions only involves thrombus suction or compression. Usual thrombus aspiration devices have limited suction volume. Therefore, we devised a method to remove the thrombus by entangling it with a stent. However, this method has some limitations. First, the stent may be wasted because a Supera stent is not deployed. Second, the handmade manual thrombus removal device cannot be advanced along the guidewire. Finally, for similar reasons, a distal embolic protection device using a filter is not possible.

The “Super GACHON” technique can be considered one of the countermeasures for thrombotic lesions if we cannot use an aspiration thrombectomy device. However, further studies are needed to ensure its safety and efficacy.

## 4. Conclusion

We presented a case of in-stent occlusion with a possible thrombotic lesion. The “Super GACHON” technique was utilized effectively and safely. This technique may be considered for dealing with thrombi lesions when an aspiration thrombectomy device cannot be used.

## Data Availability

The data that support the findings of this case shall be made available on request from the corresponding author. The data are not publicly available because of privacy or ethical restrictions.

## References

[1] Aboyans V., Ricco J. B., Bartelink M. L. E. L. (2018). Editor’s choice - 2017 ESC guidelines on the diagnosis and treatment of peripheral arterial diseases, in collaboration with the European Society for Vascular Surgery (ESVS). *European Journal of Vascular and Endovascular Surgery*.

[2] Björck M., Earnshaw J. J., Acosta S. (2020). Editor’s choice - European Society for Vascular Surgery (ESVS) 2020 clinical practice guidelines on the management of acute limb ischaemia. *European Journal of Vascular and Endovascular Surgery*.

[3] Shirai S., Hirano K., Kobayashi N. (2017). New endovascular technique for thrombus in the non-stenting zone-the Grab a Clot and Hold ON "(GACHON) technique": case report. *Catheterization and Cardiovascular Interventions*.

[4] Fukagawa T., Hirano K., Mori S. (2021). Efficacy of the novel technique HIRANODOME in preventing distal embolization during endovascular treatment of femoropopliteal lesions. *Catheterization and Cardiovascular Interventions*.

[5] Ichihashi K., Dan K. (2023). Dual retrograde tibial access thrombectomy for acute limb ischemia. *JACC: Cardiovascular Interventions*.

